# Mammalian Sirt1: insights on its biological functions

**DOI:** 10.1186/1478-811X-9-11

**Published:** 2011-05-08

**Authors:** Shahedur Rahman, Rezuanul Islam

**Affiliations:** 1 Department of Biotechnology and Genetic Engineering, Islamic University, Kushtia -7003, Bangladesh

**Keywords:** Sirt1, mammalian sirtuin, cell aging

## Abstract

Sirt1 (member of the sirtuin family) is a nicotinamide adenosine dinucleotide (NAD)-dependent deacetylase that removes acetyl groups from various proteins. Sirt1 performs a wide variety of functions in biological systems. The current review focuses on the biological functions of Sirt1 in obesity-associated metabolic diseases, cancer, adipose tissue, aging, cellular senescence, cardiac aging and stress, prion-mediated neurodegeneration, inflammatory signaling in response to environmental stress, development and placental cell survival.

## Introduction

Sirt1 (mammalian) is a member of the sirtuin family [1]. It is a nicotinamide adenosine dinucleotide (NAD)-dependent deacetylase and removes acetyl groups from many histone and nonhistone proteins [2]. Sirt1 can deacetylate a variety of substrates and is, therefore, involved in a broad range of physiological functions, including control of gene expression, metabolism and aging [1,3,4]. Sirt1 catalyzes an enzymatic reaction that generates nicotinamide and the acetyl group of the substrate is transferred to cleaved NAD, generating a unique metabolite, O-acetyl-ADP ribose [2]. The list of Sirt1 substrates is continuously growing and includes several transcription factors: the tumor suppressor protein p53, members of the FoxO family (forkhead box factors regulated by insulin/Akt), HES1 (hairy and enhancer of split 1), HEY2 (hairy/enhancer-of-split related with YRPW motif 2), PPARγ (peroxisome proliferator-activated receptor gamma), CTIP2 [chicken ovalbumin upstream promoter transcription factor (COUPTF)- interacting protein 2], p300, PGC-1α (PPARγ coactivator), and NF-κB (nuclear factor kappa B) [1-4]. In this review we will discuss some of the most relevant biological and pathophysiological functions of Sirt1 [1].

## Biological functions

### Sirt1 and obesity-associated metabolic diseases

Hepatic metabolic derangements are key components in the development of fatty liver, insulin resistance, and atherosclerosis. Sirt1 is an important regulator of energy homeostasis in response to nutrient availability. Scientists demonstrated that hepatic Sirt1 regulates lipid homeostasis by positively regulating peroxisome proliferators-activated receptor α (PPARα), a nuclear receptor that mediates the adaptive response to fasting and starvation. Hepatocyte-specific deletion of Sirt1 impairs PPARα signaling and decreases fatty acid β-oxidation, whereas overexpression of Sirt1 induces the expression of PPARα targets. Sirt1 interacts with PPARα and is required to activate PPARα coactivator PGC-1α. When challenged with a high-fat diet, liver-specific Sirt1 knockout (KO) mice develop hepatic steatosis, hepatic inflammation, and endoplasmic reticulum stress [5]. Present research data indicate that Sirt1 plays a vital role in the regulation of hepatic lipid homeostasis and that pharmacological activation of Sirt1 may be important for the prevention of obesity associated metabolic diseases [5]. Other research also shows that manipulation of Sirt1 levels in the liver affects the expression of a number of genes involved in glucose and lipid metabolism [6]. Additionally, recent studies demonstrated that modest overexpression of Sirt1 resulted in a protective effect against high fat induced hepatic steatosis and glucose intolerance [7,8]. Sirt1 orthologs also play a critical role in controlling SREBP-dependent gene regulation governing lipid/cholesterol homeostasis in metazoans in response to fasting cues. These findings may have important biomedical implications for the treatment of metabolic disorders associated with aberrant lipid/cholesterol homeostasis, including metabolic syndrome and atherosclerosis [9]. Sirt1 regulates uncoupling protein 2 (UCP2) in beta cells to affect insulin secretion. Regulation of UCP2 by Sirt1 may also be an important axis that is dysregulated by excess fat to contribute to obesity induced diabetes [10].

Sirt1 is a positive regulator of liver X receptor (LXR) proteins [11,12], nuclear receptors that function as cholesterol sensors and regulate whole-body cholesterol and lipid homeostasis. LXR acetylation is evident at a single conserved lysine (K432 in LXRα and K433 in LXRβ) adjacent to the ligand-regulated activation domain AF2 [2]. Sirt1 interacts with LXR and promotes deacetylation and subsequent ubiquitination. Mutations of K432 eliminate activation of LXRα by this sirtuin [11]. Loss of Sirt1 *in vivo *reduces expression of a variety of LXR targets involved in lipid metabolism, including ABCA1, an ATP-binding cassette (ABC) transporter that mediates an early step of HDL biogenesis [2,11]. Altogether these findings suggest that deacetylation of LXRs by Sirt1 may be a mechanism that affects atherosclerosis and other aging-associated diseases [11].

Above information suggests that Sirt1 is involved in regulation of obesity-associated metabolic diseases through regulating PGC-1α, UCP2 and LXR proteins.

### Cancer and Sirt1

**Figure 1:**
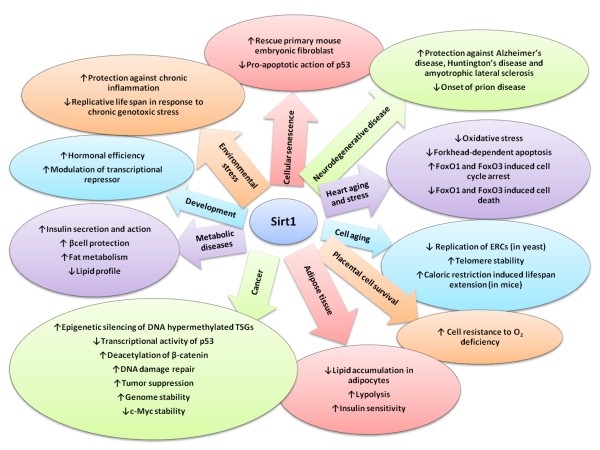
**Activation and inhibition of many cellular processes by Sirt1**.

It has been shown that Sirt1 is significantly elevated in human prostate cancer [13], acute myeloid leukemia [14], and primary colon cancer [15]. Overexpression of Sirt1 was frequently observed in all kinds of non-melanoma skin cancers including squamous cell carcinoma, basal cell carcinoma, Bowen's disease, and actinic keratosis [16]. Based on the elevated levels of Sirt1 in cancers, it was hypothesized that Sirt1 serves as a tumor promoter [17]. The first evidence of Sirt1 as a tumor promoter came from experiments showing that Sirt1 physically interacts with p53 and attenuates p53-mediated functions through deacetylation of p53 at its C-terminal Lys382 residue [18,19]. In addition, two recent studies demonstrated that DBC1 (deleted in breast cancer-1), which was initially cloned from a region (8p21) homozygously deleted in breast cancer, forms a stable complex with Sirt1 and inhibits Sirt1 activity, leading to increased levels of p53 acetylation and upregulation of p53-mediated function. Consistently, knockdown of DBC1 by RNA interference (RNAi) promoted Sirt1 mediated deacetylation of p53 and inhibited p53-mediated apoptosis induced by genotoxic stress. These effects were reversed in cells by concomitant RNAi-mediated knockdown of endogenous Sirt1 [20,21]. Sirt1 is also involved in epigenetic silencing of DNA-hypermethylated tumor suppressor genes (TSGs) in cancer cells (Figure 1). Inhibition of Sirt1 by multiple approaches (pharmacologic, over expression of a dominant negative protein or short interfering RNA) leads to TSG re-expression and a block in tumor-causing networks of cell signaling that are activated by loss of the TSGs in a wide range of cancers. Furthermore, Sirt1 inhibition causes re-expression of the E-cadherin gene (in breast and colon cancer cell lines), whose protein product complexes with β-catenin, and this gene reactivation collectively may suppress the constitutive activation of the WNT signaling pathway [22]. Sirt1 acts as a critical modulator of endothelial angiogenic functions. Inhibition of endogenous Sirt1 gene expression prevents the formation of a vascular-like network *in vitro*. Overexpression of wild-type Sirt1, but not of a deacetylase-defective mutant of Sirt1 (Sirt1 H363Y) [18,19], increased the sproutforming and migratory activity of endothelial cells [23].

DNA damage-induced acetylation of p53 protein leads to its activation and either growth arrest or apoptosis. In response to DNA damage, Sirt1 binds and deacetylates the p53 protein with specificity for its C-terminal Lys382 residue, modification of which has been implicated in the activation of p53 as a transcription factor. Expression of wild-type Sirt1 in human cells reduces the transcriptional activity of p53. In contrast, expression of a catalytically inactive Sirt1 protein potentiates p53-dependent apoptosis and radiosensitivity. So Sirt1 is involved in the regulation of p53 function via deacetylation [18].

These data suggest that increased Sirt1 expression and/or activity may increase the risk of cancer in mammals by inhibiting p53 and potentially other tumor suppressor genes. Human Sirt1 repression of dihydrotestosterone (DHT) induced androgen receptor (AR) signaling requires the NAD-dependent catalytic function of Sirt1 and the AR lysine residues deacetylated by Sirt1.

Sirt1 inhibited coactivator-induced interactions between the AR amino and carboxyl termini. DHT-induced prostate cancer cellular contact-independent growth is also blocked by Sirt1, providing a direct functional link between Sirt1 and the AR, which is a critical determinant of progression of human prostate cancer [24].

On the other hand, other researchers analyzed a public database and found that Sirt1 expression was reduced in many other types of cancers, including glioblastoma, bladder carcinoma, prostate carcinoma and ovarian cancers as compared to the corresponding normal tissues [25]. Firestein *et al *demonstrated that overexpression of Sirt1 in APC^min/+ ^mice reduces, instead of increasing, colon cancer formation. The data demonstrated that the reduction in tumor development is caused by the ability of Sirt1 to deacetylate β-catenin and promote cytoplasmic localization of the nuclear-localized oncogenic form of β-catenin [26]. Recent investigations reported that Sirt1 plays an important role in DNA damage repair and in maintaining genome integrity (Figure 1). Analyzing Sirt1 deficient mice, Wang *et al *found that Sirt1^-/- ^embryos die at middle gestation stages, displaying increased acetylation of H3K9 and H4K16, reduced chromosome condensation, impaired heterochromatin formation, and abnormal mitosis [25]. Sirt1^-/- ^cells displayed chromosome aneuploidy and structural aberrations, conceivably originated from the continuous division of abnormal mitosis. Sirt1 deficiency also causes reduced ability to repair DNA-double strand breaks (DSBs), radiation sensitivity, and impaired DDRs characterized by diminished γH2AX, BRCA1, RAD51 and NBS1 foci formation upon γ-irradiation. Thus, Sirt1 may play a role in recruiting these proteins to DNA damage sites. Yuan *et al *demonstrated that Sirt1 play a role in tumor suppression via c-Myc-Sirt1 feedback loop which regulate c-Myc activity and cellular transformation. c-Myc binds to the Sirt1 promoter and induces Sirt1 expression. However, Sirt1 interacts with and deacetylates c-Myc, resulting in decreased c-Myc stability (Figure 1). As a consequence, c-Myc's transformational capability is compromised in the presence of Sirt1 [27]. When Sirt1 was overexpressed in thymocytes in the p53^+/- ^model, after irradiation, there was a greater mean survival and lower frequency of fatal thymic lymphomas [28], further evidences of Sirt1 as a tumor suppressor.

These data provide strong evidence regarding Sirt1 that serves as a tumor suppressor. Thus, through improving metabolic conditions by increasing Sirt1 activity, it is possible to both extend lifespan and reduce cancer risk in humans in the foreseeable future [16].

Werner syndrome is an autosomal recessive disorder associated with premature aging and cancer predisposition caused by mutations of the WRN gene. WRN is a member of the RecQ DNA helicase family with functions in maintaining genome stability [29]. Sirt1 interacts with WRN both *in vitro *and *in vivo*; this interaction is enhanced after DNA damage. WRN can be acetylated by acetyltransferase CBP/p300 and deacetylated by Sirt1 WRN acetylation decreases its helicase and exonuclease activities: Sirt1 can thus reverse this effect.

Moreover, WRN acetylation alters its nuclear distribution. Down-regulation of Sirt1 reduces WRN translocation from nucleoplasm to nucleoli after DNA damage. This suggests that Sirt1 regulates WRN-mediated cellular responses to DNA damage through its deacetylation and can help to prevent cancer [29].

The role of Sirt1 in cancer is still in controversy, it could act as both tumor suppressor or tumor promoter. This may depend on its targets or the cellular location or specific cancers.

### Adipose tissue and Sirt1

Adipose tissue recently emerged as a pivotal organ controlling lifespan. Genetic manipulations aiming at modifying fat mass also impact on the duration of life in several model organisms [30]. Sirt1 represses peroxisome proliferator-activated receptors gamma transactivation and inhibits lipid accumulation in adipocytes (Figure 1). The effect of adipose tissue reduction on lifespan could be due to the production of adipokines acting on target tissues such as the brain, or due to the indirect prevention of age-related metabolic disorders like type 2 diabetes or atherosclerosis [31]. Sirt1 improves insulin sensitivity, which has implications toward resolving insulin resistance and type 2 diabetes [32] (Figure 1).

Sirt1 represses PPARγ by docking with its cofactors NCoR (nuclear receptor corepressor) and SMRT (silencing mediator of retinoid and thyroid hormone receptors). Repression of PPARγ by Sirt1 is also evident in 3T3-L1 adipocytes, where overexpression of Sirt1 attenuates adipogenesis, and RNA interference of Sirt1 enhances it. In differentiated fat cells, upregulation of Sirt1 triggers lipolysis and loss of fat [33] (Figure 1). Sirt1 is a functional regulator of PGC-1α that induces a metabolic gene transcription program of mitochondrial fatty acid oxidation [24].

### Sirt1 and aging

Sirt1 and its ortholog appear to have a key role in determining lifespan of yeast, flies, and mice [34-36]. One mechanism by which Sirt1 extends lifespan in the budding yeast, *Saccharomyces cerevisiae*, is by modifying the physical structure (deacetylation) of key heterochromatin regions of the genome, especially extrachomosomal rDNA circles (ERCs), which accumulate in the nucleolus [34,37,38]. This results in suppression of replication of ERCs during each cell division (Figure 1). Budding yeasts divide by asymmetric self-renewal where daughter cells receive primarily newly synthesized organelles, while mother cells preferentially retain "old" organelles [37,38]. ERCs accumulate in mother cells in the nucleolus as the mother cell continues to divide until 500 to 1000 copies of ERCs are present in the nucleolus, usually after about 20 divisions. Then the nucleolus disintegrates and mother cells no longer grow/divide and become quiescent. Thus, yeast mother cells have lifespan of approximately 20 divisions. Yeast cells containing an extra copy of the Sirt1 gene accumulate ERCs more slowly because extra Sirt1 silent the rDNA region, suppressing their formation and accumulation allowing more divisions and longer lifespan than wild-type yeast [34,37,38].

In human stem cells ageing is associated to telomere erosion. In the absence of telomerase, telomere shortening occurs and eventually causes a change in chromatin structure. This is recognized by the DNA-damage response pathway and activates programmed cell death. Sirt1 could have a role in telomere maintenance during stem cell aging because of its essential role in gene silencing [38]. Yeast SIR proteins are associated with telomeres [38-40]. Maintenance of telomere stability via histone deacetylation and chromatin stability modifications is also a potential mechanism by which Sirt1 could influence stem cell aging and ultimately contribute to longer lifespan [41] (Figure 1).

Another way Sirt1 could be involved in stem cell aging via its central role in caloric restriction (CR)-induced lifespan extension [42,43] and its link to age-related reactive oxygen species (ROS) generation [44-46]; both are highly dependent on mitochondrial metabolism. ROS can damage macromolecules and lipids [47] and accumulation of these damaged molecules over time is believed to result in age-related decline in cell and tissue function. The primary source of ROS in cells is mitochondria. ROS are a by-product of ATP generation via the electron transport chain (ETC) and oxidative metabolism. They are toxic and damaging at high concentrations, but they are also essential for proper oxygen sensing, maintenance of cellular redox state [48,49], and regulation of proliferation and differentiation [50-52] at lower concentrations. CR, known to extend lifespan in several model organisms including yeast, flies, worms, and rodents [42,46], appears to decrease ROS generation in several mouse tissues by forcing mitochondrial reprogramming to preferentially generate ATP by β-oxidation of fatty acids instead of carbohydrate catabolism. Sirt1 has been implicated in CR-induced lifespan extension in mice (Figure 1) because Sirt1 gene deleted mice fail to display at least some of the phenotypes of CR mice [53], the most important of which is failure of CR to extend lifespan [54]. Also, Sirt1 transgenic overexpressing mice have a CR-like phenotype [55]. Furthermore, CR upregulates Sirt1 in several mouse tissues and, importantly, in human muscle tissue [56-58].

### Sirt1 and cellular senescence

Cellular senescence is a state of permanent cell cycle arrest, accompanied by defined morphological changes that may be induced by several stimuli. Under certain conditions, Sirt1 localizes to discrete nuclear substructures with PML (promyelocytic leukaemia) protein to form nuclear bodies. PML may co-activate or corepress various transcription factors localized in the nuclear bodies, thus mediating different apoptotic signals. Up-regulation of a PML isoform, PML-IV, recruits Sirt1 into the nuclear bodies together with p53. Thus, Sirt1 rescues primary mouse embryonic fibroblast from PML-mediated premature cellular senescence by inhibiting the pro-apoptotic action of p53 [59] (Figure 1). Mouse embryonic fibroblasts lacking Sirt1 had an extended replicative potential and showed greater proliferative capacity following chronic but not lethal stress, a phenotype shown to require the tumor suppressor p19ARF and its downstream target p53 [50,60]. A recent study showed that Sirt1 levels are lower in cells that have been serially split or in dividing tissues of aged mice, such as thymus and testis, but not in immortalized cells or post-mitotic organs [61].

### Sirt1 in heart aging and stress

The prevalence of heart diseases, such as coronary artery disease and congestive heart failure, increases with age. Recent findings from transgenic mice with cardiac-specific overexpression of Sirt1 demonstrated a delayed aging and protection against oxidative stress in the heart [62]. Low to moderate (about 3-fold to 8-fold) Sirt1 overexpression efficiently protected mice from paraquat-induced cardiac stress and apoptosis, and delayed the onset of age-dependent heart dysfunctions. Conversely, greater increases in Sirt1 levels (about 13-fold) induced oxidative stress and apoptosis, ultimately leading to cardiomyopathy and decreased survival [63]. Several other studies in mice models further demonstrated a protective role of Sirt1 against hypertrophic and oxidative stresses [64-66] (Figure 1). Loss of cardiomyocytes through apoptosis has been proposed as a cause of ventricular remodeling and heart failure. Ischemia and hypoxia-induced apoptosis of cardiomyocytes reportedly plays an important role in cardiac pathologies. Exposure of the cells to resveratrol caused rapid activation of Sirt1, which had a dual effect on FoxO1 function: Sirt1 increased FoxO1's ability to induce cell cycle arrest, but inhibited FoxO1's ability to induce cell death (Figure 1). This effect could be reversed by Sirt1 inhibition. Results indicated that resveratrol inhibits hypoxia-induced apoptosis via the Sirt1 FoxO1 pathway in H9c2 cells. This polyphenol may have potential in preventing cardiovascular disease, especially in coronary artery disease (CAD) patients [67]. Sirt1 also had a dual effect on FoxO3 function: Sirt1 increased FoxO3's ability to induce cell cycle arrest and resistance to oxidative stress but inhibited FoxO3's ability to induce cell death (Figure 1). Thus, one way through which members of the Sir2 family of proteins may increase organismal longevity is by tipping FoxO dependent responses away from apoptosis and toward stress resistance [51]. Sirt1 also deacetylates and represses the activity of the FoxO3a and other mammalian forkhead factors. This regulation appears to be in the opposite direction in respect to the genetic interaction of Sir2 with forkhead in *C. elegans*. By restraining mammalian forkhead proteins, Sirt1 also reduces forkhead-dependent apoptosis [68].

### Sirt1 in prion-mediated neurodegeneration

Sirt1 plays a protective role in several models of neurodegenerative disease. Sirt1 may serve as a downstream effector of increased NAD biosynthesis and delay axonal degeneration in a mouse model of Wallerian degeneration [69]. Importantly, overexpression of Sirt1 also protects against Alzheimer's disease, Huntington's disease and amyotrophic lateral sclerosis in various model systems [70-73] (Figure 1), consistent with its proposed neuroprotective function. Prion diseases are unique among neurodegenerative diseases in that they are transmissible while still sharing commonalities with other neurodegenerative diseases such as the accumulation of aggregates of misfolded protein, a prominent astrocytic and microglial response, and loss of neurons in the central nervous system [74]. Research report show that the onset of prion disease is delayed by CR and in the Sirt1 KO mice fed ad libitum (Figure 1). CR exerts no further effect on the Sirt1 KO strain, suggesting the effects of CR and Sirt1 deletion are mechanistically coupled. In conjunction, Sirt1 is downregulated in certain brain regions of CR mice [75].

Sirt1 inhibition increased acetylation and decreased phosphorylation of IRS-2; it also reduced activation of the Ras/ERK1/2 pathway, suggesting that Sirt1 may enhance IGF-I signaling in part by deacetylating IRS-2 [76]. Either the inhibition of Sirt1 or of Ras/ERK1/2 was associated with resistance to oxidative damage. Markers of oxidized proteins and lipids were reduced in the brain of old Sirt1 deficient mice, but the lifespan of the homozygote knockout mice was reduced under both normal and calorie-restricted conditions [76]. These findings are consistent with findings in *S. cerevisiae *and other model systems, suggesting that mammalian sirtuins can play both protective and proaging roles [76].

### Sirt1 and inflammatory signaling in response to environmental stress

Researchers have determined that Sirt1 provides protection against chronic inflammation by controlling the acetylation of nuclear factor kappa B (NF-κB), a transcription signaling pathway involved in the innate immune response (Figure 1). Generation of myeloid-specific Sirt1 knockout mouse model (Mac-Sirt1 KO) and demonstrated that the absence of Sirt1 resulted in hyperacetylated NF-κB, which led to an increase in the transcription of proinflammatory genes in macrophages [77]. Using bone marrow-derived macrophages from wild-type and Mac-Sirt1 KO mice, the investigators confirmed their results *in vitro *[77]. The Mac-Sirt1 KO cells also exhibited hyperacetylated NF-κB levels [77].

Taken together, the above findings demonstrate Sirt1 as an important mediator between environmental stress and immune system activation [77]. Sirt1 also limits replicative lifespan in response to chronic genotoxic stress [50] (Figure 1).

### Development and Sirt1

High levels of Sirt1 mRNA are detectable in heart, brain, spinal cord and dorsal root ganglia of embryos, indicating that it may play a critical role in development [78]. Consistent with this, Sirt1 knockout mice show developmental defects. About half of the numbers of pups expected are typically born, and of these approximately 20% survive to adulthood. Sirt1 knockout mice are also markedly smaller than their littermates, develop more slowly and show dramatic perturbations in eye morphogenesis and cardiac septation [79]. Both males and females that survive to adulthood are sterile, males have low sperm counts and females fail to ovulate, which is probably due to a hormonal inefficiency [79,53] (Figure 1). Sirt1 KO models differ enormously in their phenotype accordingly to the genetic background. Their lifespan ranges from few weeks to two years [65].

Explanations for these phenotype of the knockouts may come from biochemical studies. The Hairy and Hey subfamilies of the bHLH (basic helix-loop-helix) proteins primarily function as transcriptional repressors that direct metazoan development [80]. The mammalian homologue of *Drosophila Hairy *is Hes1 and of *Hey *is Hey2. Sirt1 interacts with Hes1 and Hey2, both *in vivo *and *in vitro*, and modulates their ability to repress target promoters [1]. Sirt1 also interacts with mammalian and chicken protein BCL11A and CTIP2, respectively, which are involved in haematopoietic cell development and malignances [81].

### Sirt1 in placental cell survival

Sirt1 is upregulated in primary trophoblasts from human placenta exposed to hypoxia and enhances the expression of the NDRG1 (N-Myc down-regulated gene 1), which inhibits p53 transcription. Therefore by direct inactivation of p53 or by reducing its expression via NDRG1, Sirt1 promotes cell resistance to oxygen deficiency in the placenta [1] (Figure 1). Moreover, the expression of NDRG1 in hypoxia was reduced by sirtinol, which inactivates Sirt1 [66]. Neither overexpression nor silencing of NDRG1 influenced Sirt1 expression. These results provide further support to the protective function of NDRG1 in hypoxia, implicating Sirt1/p53 signaling in the process [82].

## Perspectives

When Sir2 was first discovered in yeast 27 years ago, it could hardly have been anticipated how much interest would be taken in this family of proteins [1]. Several studies over the last 10 years have illuminated our understanding of the role of sirtuins in different organisms. Currently, biological functions of Sirt1 range from DNA repair to aging. Yet many questions remain to be answered. Further research will give us a far clearer perspective as to whether the sirtuins may provide novel therapies to alleviate age-associated changes, such as diabetes, cancer and cardiovascular disease, and possibly extend healthy human lifespan.

## Competing interests

The authors declare that they have no competing interests.

## Authors' contributions

SR and RI contributed to the preparation of the manuscript and approval of its final version.
